# Achieving health equity in cancer care in the Philippines

**DOI:** 10.3332/ecancer.2023.1547

**Published:** 2023-05-10

**Authors:** Rey Arturo T Fernandez, Frederic Ivan L Ting

**Affiliations:** 1 Ateneo Professional Schools, Graduate School of Business, Rockwell Drive, Makati 1210, Philippines; 2 Division of Oncology, Department of Internal Medicine, Corazon Locsin Montelibano Memorial Regional Hospital, Bacolod 6100, Philippines; 3 Department of Clinical Sciences, College of Medicine, University of St. La Salle, Bacolod 6100, Philippines

**Keywords:** cancer care, health equity, Filipinos

## Abstract

Notwithstanding the progress made across the cancer care continuum, a major problem that many patients with cancer experience is the difficulty of access to global standards of care. Awareness of this problem has been increasing most especially when the economic context of a country forces health systems to deliver quality care despite the rising costs of diagnostic and therapeutic innovations amidst limited resources. Ultimately, inappropriate delivery of care to patients with cancer contributes to inadequate and unequal access to high-value therapy increasing financial toxicity among patients. This paper aims to highlight (1) the economic burden of cancer in the Philippines, (2) the saliency of identifying low-value interventions which come in two forms: the persistent over usage of proven ineffective modalities, and the underusage of potentially effective ones, and (3) the adverse effects of a decentralized health care system. The paper will also provide suggestions to address the challenges of achieving health equity in cancer care.

## Introduction

A growing number of patients are diagnosed with cancer each year. As cancer incidence rises, health expenditure in cancer therapy increases. This results in variable access and clinical outcomes predominantly affecting low- to middle-income countries (LMICs) wherein government funding for cancer care is limited. When public access to high-value therapy is inadequate, anticancer treatment is often delayed, prematurely terminated, or in some cases never started due to financial toxicities imposed by rising cancer drug prices. Consequently, this leads to poor patient outcomes and a loss of economic productivity adversely affecting the overall health ecosystem further worsening the already burdensome financial challenges in a majority of patients diagnosed to have cancer.

Notwithstanding the progress made across the cancer care continuum, a major problem that many patients with cancer experience is the difficulty of access to the global standards of care. As with other medical fields, the practice of oncology is not immune to the persistent problem of both underuse and overuse of diagnostic modalities and therapeutic interventions. Awareness of this problem has been increasing most especially when the economic context of a country forces health systems to deliver quality care despite the rising costs of diagnostic and therapeutic innovations amidst limited resources. Ultimately, inappropriate delivery of care to patients with cancer contributes to inadequate and unequal access to high-value therapy increasing financial toxicity among patients.

**Figure 1.**
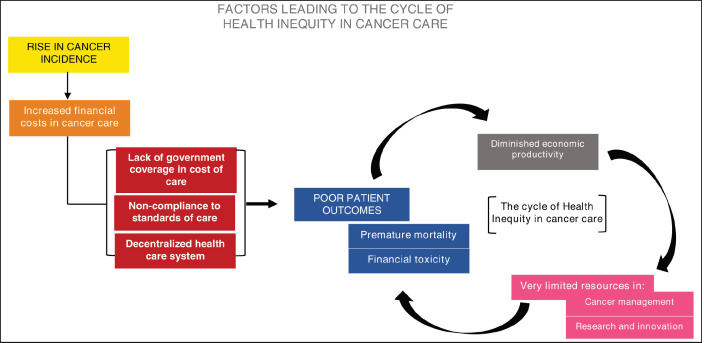
Increased financial costs in cancer care are exacerbated by limited access, inappropriate management, and a decentralized health care system leading to a detrimental cycle of poor patient outcomes, diminished economic productivity and resources.

Following the decentralization of the Philippine health care system, health decisions that were made in the national level were transferred to local government units (LGUs). The initiative to increase independence of the local government was to ensure that the unique needs of each region are properly addressed. However, the increased autonomy of the local government from the 1991 decentralization, reinforced by the recent Mandanas-Garcia ruling which fully transfers or devolves the delivery of basic services to LGUs, has contributed to health inequities including the management of patients with cancer (Figure 1).

In this context, the objectives of this paper are to highlight (1) the economic burden of cancer in the Philippines, (2) the saliency of identifying low-value interventions which come in two forms: the persistent over usage of proven ineffective modalities, and the underusage of potentially effective ones, and (3) the adverse effects of a decentralized health care system. The paper will also provide suggestions to address the challenges of achieving health equity in cancer care.

## The burden of cancer and its effect on the Philippine health system

The burden of cancer is continuously growing worldwide [30]. As cancer incidence rises, its socioeconomic impact becomes more apparent. Global annual spending on oncology drugs has increased from $75 billion in 2010 to $150 billion in 2018 [17]. With a growing population, chronic diseases like cancer will inevitably overwhelm health care systems – most especially in LMICs where access to novel and high-value treatment are limited. The already very low budget of the Philippine government for cancer care will be inadequate to address the needs of a growing Filipino population affected by a highly prevalent and debilitating disease. As of 2021, the current Philippine gross domestic product spent for health consumption is only at 6% [24].

In the cancer care continuum, selection of treatment is the most basic and at the same time the most complicated step and thus requires the expertise and collaboration of a multidisciplinary team of specialists [9] since modern treatment options involved multimodality regimens including surgery, radiotherapy, chemotherapy and targeted therapy including small molecule inhibitors and immunotherapy [27]. As cancer treatment has become more and more personalized leading to better treatment outcomes [3], the cost of care has been steadily increasing rendering these novel treatment options inaccessible to a great majority of Filipinos [6, 22].

Due to the high costs of cancer therapy, patients strongly depend on financial assistance to fund their medical needs. In response, the Philippine government has released a 620-million-peso cancer assistance fund for the year 2021 and plans to allot 786 million Philippine pesos for the year 2022 [31]. The funding was mandated through the National Integrated Cancer Control Act which was signed into law last 2019. Although a major accomplishment, the fund allocated remains inadequate to support Filipinos diagnosed with cancer.

**Figure 2.**
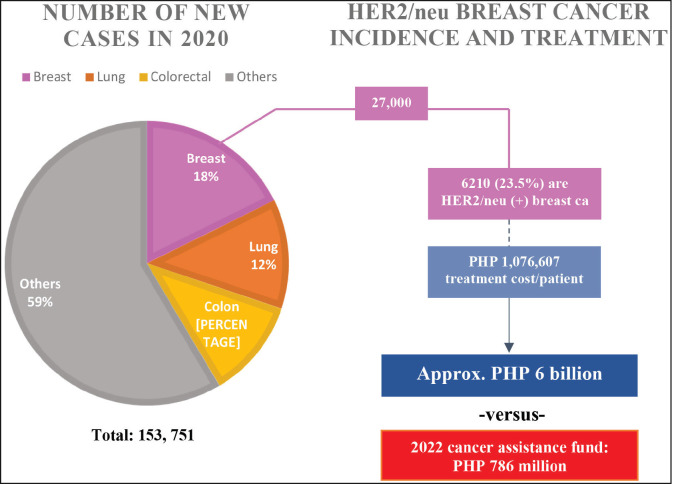
Number of new cancer cases in the Philippines in the year 2020, HER2/neu breast cancer incidence and treatment costs.

The World Health Organization (WHO) International Agency for Research on Cancer reported that there were 153,751 newly diagnosed cases in year 2020; majority of which are breast, lung and colon carcinomas [30]. To understand the implications of the cost of anticancer therapy, breast cancer treatment can be taken as an example. Breast malignancies, the leading cause of cancer among Filipinos, represent approximately 18% of all cancer types of which 27,163 women have been diagnosed in the year 2020 alone. HER2/neu-positive breast cancer, a more aggressive subtype associated with poorer survival outcomes [14], requires a monoclonal antibody-based therapy such as Trastuzumab to target the overexpressed protein kinase [21]. Unfortunately, the cost of Trastuzumab may reach as much as PHP 1 million for the entire treatment cycle thus despite its well-established efficacy to improve patient outcomes, it cannot be afforded by low and middle-income Filipinos [23] since approximately 23.7% of the population lived below the national poverty line [4]. Of the 27,000 Filipino women diagnosed with breast cancer, about 23.5% are HER2/neu positive [11]. With 6,210 HER2/neu – positive patients requiring Trastuzumab, the government needs to raise more than PHP 6 billion to shoulder treatment costs. The amount is a far cry from the 2022 budget which is intended to cover all cancer types (Figure 2). Thus, health equity in cancer care is very difficult to achieve due to the financial constraints that limit patient access to ideal therapy.

Despite antineoplastic treatment being costly, the saliency of cancer funding is apparent when productive years of life are lost from premature death due to malignancies. The Global Burden of Diseases 2019 study enabled the prospective assessment of cancer burden in terms of cancer incidence and disability-adjusted life years (DALYs) through a location and time-specific registry [12]. The systemic analysis measured these parameters in the light of the Sociodemographic Index (SDI) quintiles of the territories it covered. Countries were grouped into quantiles based on their SDI values. High quantile countries include United States of America and Germany while the Philippines belongs to the middle quantile. The study revealed that while territories in the high SDI quantile had the highest number of new cases in 2019, countries from middle SDI quantile had the highest number of cancer deaths and DALYs reflecting years lost due to premature death and disability. Although DALYs are not an economic marker, it is a time-based measure that takes into account years of healthy life lost due to premature mortality and disability [35]. Since patients with cancer become less economically productive, this loss of productivity, in a larger scale, affects overall health economics. Therefore, to prevent potential economic losses due to premature death, a systematic assessment of cancer funding is paramount wherein cost and value of therapy are of prime consideration. This assessment also encompasses prioritizing high-value treatment.

## Cancer care and its financial burden

The treatment modalities of cancer are costly; they increase the risk of financial catastrophe for Filipinos. During this time of personalized medicine, prognosis of patients with late-stage cancers have significantly improved. Compared with conventional chemotherapy, new generation agents such targeted treatments and immunotherapies have increased over-all and disease-free survival while minimizing chemotherapeutic toxicities and improving quality of life [26]. However, there are important issues with the use of these newer treatments.

First, not all novel therapies are of high clinical value [25]. Oncology drug prices correlate poorly with clinical benefit with some drugs providing less benefit – even more harm – to patients compared to conventional chemotherapy relative to their market costs [17]. Although Food and Drug Administration (FDA)-approved cancer medications demonstrate statistical significance compared to placebo, this does not equate to meaningful benefits for patients most especially if the statistical difference is small and the costs are high. A statistically significant prolongation of overall survival may be demonstrated even if the drug being investigated only improves life span by a few weeks [28]. Hence, newly approved anticancer drugs should undergo surveillance to evaluate their cost-effectiveness relative to other oncologic medications in the market. A higher number of funded anticancer drugs does not also equate to better clinical outcomes for patients. An analysis of oncologic medicines in Australia and New Zealand concluded that out of the 35 cancer medicines funded in Australia alone, only 3 provided a meaningful benefit to patients with malignancies [10].

Second, novel agents are often protected by patent which enable pharmaceutical companies to dictate the initial market price. Without competition, the demand for effective therapies remains high driving prices upward. Furthermore, the impact of free market forces are minimal on cancer drug prices because of the demand and willingness of patients to pay due to the morbidities associated with cancer [17]. To improve affordability, regulatory groups advocate the development of biosimilar agents which are structurally similar with the original patented biologic drug. These drugs are also clinically effective hence can stimulate market competition. However, to gain regulatory approval and consumer use, these agents have to undergo rigorous clinical studies to demonstrate efficacy requiring substantial amount of funding and resource allocation. Moreover, generics also experience delay in market entry challenged by regulatory hurdles and drug manufacturer patents [15].

These financial challenges especially in LMICs highlights the importance of cancer screening to facilitate early detection of cancer which leads to better patient outcomes and possible lower costs of treatment[5, 18]. Unfortunately, a large sum of the budget for cancer care is allocated on the curative than the preventive arm [7]. On the other hand, overutilization of screening modalities are also costly. As an example, the addition of breast ultrasonography on top of mammography may increase the sensitivity of detecting early-stage breast cancer but has a limited impact on breast cancer mortality and quality of life years gained [29]. Supplemental screening with ultrasonography for breast cancer may increase detection rates but likewise increases false-positive results [19]. False-positive results may also further augment the financial burden by subjecting patients to unnecessary work-ups. Despite evidence suggesting higher detection rates for invasive breast cancers [37], the effects of supplemental screening with ultrasonography on breast cancer outcomes remain unclear. The diagnostic work-up of a patient with cancer is also associated with increased indirect costs including transport costs. This includes gaining access to financial support systems from government institutions and insurance companies. Despite the evidence that insurances provide protection against catastrophic diseases, there has been no significant impact of having insurance on financial catastrophe among Filipino patients with cancer [23]. Likely culprits raised were inadequate benefit package or a lack of support value or very low coverage by the insurance company. Prioritizing which treatment modalities to fund for public access, carefully selecting and using health technologies for early detection and education, and evaluating the impact of insurance coverage highlight the importance of proper budget allocation in cancer care. However, a substantial hindrance arises from the current state of the health care system of the Philippines.

## Decentralization of health care in the Philippines

In 1991, the Philippine government introduced a devolution of national government services including the health sector [13]. The main purpose was to empower LGUs in addressing the specific needs of their local community. The devolution involved the transfer of equipment, records and assets of the Department of Health (DOH) to the LGUs [8]. Prior to the devolution, DOH recognized that many LGUs faced material, financial and human resource constraints. Without a strategic plan, the devolution resulted in a decline in the quality of health care delivery particularly in remote and rural areas due to high dependence on central level support. Some areas were not equipped with tertiary health care facilities. Furthermore, there was difficulty in managing referral systems across political or administrative units. These were further exacerbated by the mismatch between the cost of the devolved functions and revenue allotment.

The disparity in public health access among LGUs is still seen at present. The COVID-19 pandemic highlighted this disparity when hospitals across the country, most especially in the capital, were overwhelmed with the sheer number of patients who required tertiary care. In response, hospitals tasked all government physicians, regardless of field of training, to go on duties in triage units and COVID-19 wards [20]. Therefore, patients with non-COVID-19 related diseases, like cancer, who warranted specialized care, were greatly affected for their medical needs suffered by the lack of and displaced human resource brought about by the pandemic. Patients who were most affected were the ones who lived outside regions with specialized health care like Metro Manila [32]. Due to the paucity of specialized care in rural areas and imposed travel restrictions, patients with cancer had delayed access to ideal management. A decentralized health system, originally intended to strengthen LGUs response to their local communities, left the country ill-equipped for the COVID-19 pandemic. The Philippine’s primary health care system failed to serve as the primary line of defense [2]. Although the Philippine Society of Medical Oncology acted quickly to address the displaced need for treatment of patients with malignancies [33, 34], the pandemic exposed vulnerabilities in health care delivery such as cancer management.

The devolution of the health care system also led to a decentralized decision-making process. As functions were transferred to the LGUs, health decisions were mostly made by local mayors and governors [16]. The technical decision-making skills required in fulfilling national guidelines mandated by DOH are inadequate in highly politicized health projects. Politicization of decision-making pose challenges in resource management and health service delivery. Careful planning in upgrading health facilities, hiring qualified professionals, and implementing health promotion initiatives were made by the mayor or governor in some areas and not by the local health officer. Moreover, without proper endorsement from one political term to the next, government spending for prior health projects become futile. This may be brought about by disinterest of the succeeding politician or a lack of alignment with the national government’s health care agendas. The devolution of the healthcare system is not by itself a barrier in cancer care because it primarily enables LGUs to respond to the local needs of their communities. Yet, it is essential to recognize the challenges and pitfalls of a decentralized system.

Altogether, inappropriate management despite limited resources in a country with a vulnerable health care system augments the growing financial burden imposed by a rising cancer burden. These factors are barriers in achieving health equity in cancer care, hence, they need to be addressed.

## Achieving health equity in cancer care

**Figure 3.**
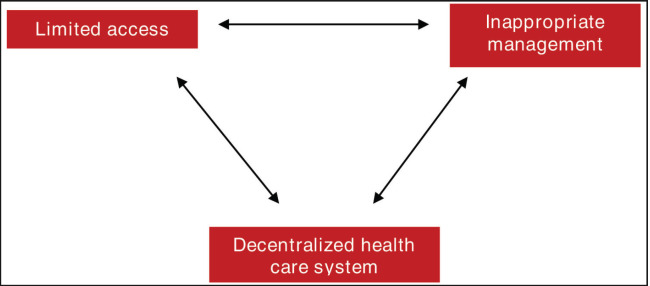
Interconnectedness of the three main challenges in achieving health equity in cancer care.

Interventions proposed to solve financial problems are usually straightforward. A lack of budget is resolved by increasing funding. Other means to potentially reduce costs indirectly is through outsourcing. However, the financial burden due to increasing cancer costs and incidence is not resolved by simply allocating more funds for the management of patients with cancer. It requires a systematic approach to address the three main barriers identified, namely, lack of access, inappropriate management and a decentralized healthcare system (Figure 3). Since these factors are interconnected, interventions implemented in one aspect will affect the other. On the other hand, solutions proposed for one of the three barriers may not be effective if the other factors are not addressed concurrently. How these factors relate with one another is illustrated below in Figure 3. Limited access due to financial constraints are exacerbated by inappropriate management thus reducing allocation for high-value therapy. Limited resources is likewise an additional challenge to appropriate management since funding is essential in the research and evaluation of the cost-effectiveness of interventions. Meanwhile, a decentralized health care system affects both the access to funds and the prioritization of health interventions most especially from a highly politicized governance. In turn, constrained resources reduce the ability of an institution to implement health projects. Moreover, inappropriate management further contributes to the reduction of the availability of financial support for cancer care.

**Figure 4.**
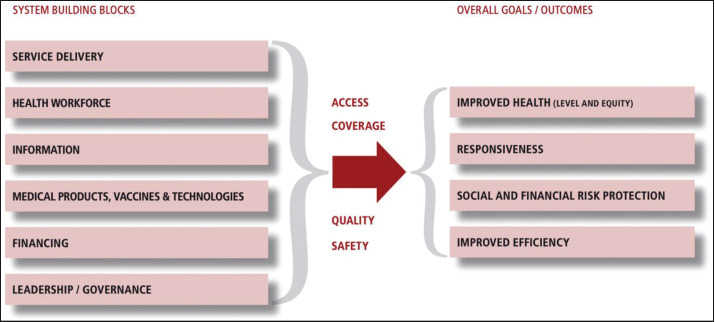
The WHO Health System Framework. Retrieved from strengthening health systems to improve health outcomes – WHO’s Framework for Action.

Awareness of how these factors relate with each other is salient for an institution to arrive to a systematic solution [1]. This awareness is a step towards understanding the importance of generating knowledge to guide policy and decision making. A framework that can guide institutions in addressing the three main challenges of achieving health equity in cancer care is the WHO Health System Framework (Figure 4) which is currently being used as a catalyst for achieving global health targets [36]. The framework’s main purpose is to address the need to improve the performance of health systems which are composed of institutions, people and resources. The rationale behind improving health systems stems from its inability to match the development of sophisticated and advanced interventions for curing diseases like cancer. This inability continues to widen the gap in health outcomes of patients suffering from the disease. Strengthening the six system building blocks would address health inequities in cancer care. Good health services which deliver effective and safe health interventions and a responsive health workforce are essential in every LGU health project to minimize waste of resources and achieve the best possible health outcomes. Data gathered from a functioning health information system would empower both national and LGUs to ensure timely and efficient interventions. Information generated would allow proper health technology assessment in order to prioritize cost-effective interventions. Improving the access to medical products and technologies as well as good health financing to raise funds for health care services would protect patients with cancer from financial catastrophe. Finally, good governance enables the development of policies that promote innovation and accountability. This would also reinforce the capacity to guide and intervene in the use of health services in clinical practice to eliminate low-value management by withdrawing fund allocation.

## Conclusion

After identifying the different factors leading to health inequity in the Philippines such as (1) economic burden of cancer in the Philippines, (2) the saliency of identifying low-value interventions which come in two forms: the persistent over usage of proven ineffective modalities, and the underusage of potentially effective ones, and (3) the adverse effects of a decentralized health care system, it is truly imperative that the Philippines addresses these challenges to achieve health equity in cancer care.

## Financial declaration and conflicts of interest

Both authors have no conflicts of interest to declare, and no funding was provided for this article.
